# A hard day’s night: Patterns in the diurnal and nocturnal foraging behavior of *Apis dorsata* across lunar cycles and seasons

**DOI:** 10.1371/journal.pone.0258604

**Published:** 2021-10-22

**Authors:** Allison M. Young, Sangamesh Kodabalagi, Axel Brockmann, Fred C. Dyer

**Affiliations:** 1 Department of Integrative Biology, Michigan State University, East Lansing, MI, United States of America; 2 Ecology, Evolution, and Behavior, Michigan State University, East Lansing, MI, United States of America; 3 National Centre for Biological Sciences, Tata Institute of Fundamental Research, Bangalore, Karnataka, India; 4 Department of Apiculture, University of Agricultural Sciences, GKVK, Bangalore, Karnataka, India; Universitat Leipzig, GERMANY

## Abstract

The giant honey bee *Apis dorsata* is unusual in being able to forage during both the day and the night. To date, the extent of this unique nocturnal foraging behavior and the environmental factors correlating with it have not been deeply investigated. We conducted the first systematic investigation into the nocturnal behavior of *A*. *dorsata* in Southern India by tracking the daily and nightly foraging activity of *A*. *dorsata* colonies in an urban environment for 8 months, over multiple seasons and lunar cycles. We found strong evidence that *A*. *dorsata* can behave in a manner that is “cathemeral” (active over the entire diel cycle) when environmental illumination is sufficient for nocturnal flight. However, workers were not always active even when the environment should have been bright enough for them to forage, suggesting that their nocturnal foraging behavior was also affected by seasonal changes in resource availability. The foraging activity observed during the day versus twilight versus night differed between seasons; notably, nocturnal activity rates were higher than diurnal activity rates during the winter. We found that at our study site *A*. *dorsata* routinely exhibits both diurnal and crepuscular activity, foraging just as intensely during the short twilight hours as during the day. The high foraging activity observed during the twilight and nighttime hours shows that *A*. *dorsata* colonies can extend their foraging beyond the daylight hours and reveals that foraging during these dimly lit hours is an integral part of their foraging ecology. This evidence of the importance of nocturnal and crepuscular foraging by *A*. *dorsata* paves the way for future studies examining the role of this species in nocturnal pollination networks, the contribution of nocturnal foraging to colony-level nutrition and energy budget, and the evolution of this unusual behavior. Future work comparing nocturnal activity in light polluted urban environments versus unpolluted natural environments is particularly encouraged to determine the generalizability of these findings.

## Introduction

Most animal species have evolved to be active only during limited periods of the 24-hour cycle (i.e., the diel cycle). Those periods of activity, often referred to as the species’ temporal niche, are usually tightly controlled by the endogenous circadian clock, which has been entrained by environmental cycles such as the light-dark cycle, temperature, humidity, etc. [1–3]. An animal’s temporal niche presumably evolved to maximize fitness; animals are generally both adapted and limited to their temporal niche through internal (e.g., anatomy, physiology, sensory and thermoregulatory capabilities [1, 2, 4]) and external (e.g., biotic and abiotic environmental conditions, predation [2, 5, 6]) factors. However, animals can sometimes extend or switch their temporal niche through changes in the oscillation of their circadian system, or through direct effects of environmental factors on activity (often referred to as ‘masking’), which occurs when environmental factors such as illumination or temperature inhibit or stimulate an animal’s behavior in a way that overrides their circadian clock [1–3, 7].

Although much work to date has studied temporal niches and temporal niche switching using mammalian species [2,5, 7, but see 3], these phenomena are found throughout the animal kingdom. In bees, for example, evidence that species are adapted to be active only during certain portions of the diel cycle are shown through adaptations in anatomy (e.g., body size, eye morphology and anatomy, ocelli size [8–12]) and physiology (e.g., visual sensitivity and resolution, spatial photon summation, thermoregulation [8, 12–15]). A notable exception to the general rule that animals are restricted to only one part of the daily light-dark cycle is the behavior of the giant honey bee, *Apis dorsata*. This species, which lives throughout tropical Asia, is highly unusual among bees in being able to actively fly and forage during the day as well as some nights [16, 17].

The ability of *A*. *dorsata* to extend its temporal niche into the night is likely due in part to traits associated with their large size. As the second largest honey bee species in body size, *A*. *dorsata* has correspondingly large eyes, as well as more and larger-sized ommatidia, which is correlated with better visual sensitivity in dim light [9, 11, 18]. However, large body size alone is not sufficient for nocturnal activity: the largest honey bee species, *A*. *laboriosa*, does not forage at night, though this might be due in part to the colder nocturnal temperatures it experiences living in higher altitudes [19–22]. The heads of *Apis dorsata* workers also have a raised vertex so their ocelli are more pronounced, improving their light sensitivity [23, 24], as larger, more pronounced ocelli are typically associated with dim-light species [25]. There is also evidence that *A*. *dorsata* has neural circuitry in the optic lobes that perform photon summation across wide parts of their visual field, which improves light capture and would allow them to discriminate coarse images in moonlight even if the resolution of those images would be lower [13, 26]. None of these factors can fully explain their ability to fly at night, however, as the eyes of *A*. *dorsata* are thought to be much less sensitive than those of bee species that are specialized for crepuscular or nocturnal activity [11]. Apart from vision, another way in which large body size could permit nocturnal activity in an endothermic insect like a honey bee would arise from the ability to maintain a higher body temperature, and thus sustain flight at lower ambient temperatures [27, 28]. However, *A*. *dorsata* has been shown to deviate from the expected size-related patterns in flight energetics and is actually less able to fly at low ambient temperatures than the smaller species *A*. *cerana* and *A*. *mellifera* [29]. Taking all of this evidence into account, visual adaptations are likely to be the most important factor accounting for their ability to fly at night, even if puzzles remain about the visual adaptations that support nocturnal flight.

Behavioral evidence supports the hypothesis that some level of illumination is necessary for *A*. *dorsata* foragers to be nocturnally active. Reports show that worker bees can actively forage throughout the night if the moon is at least half full [11, 16]. Furthermore, in cities, *A*. *dorsata* may be capable of flying using artificial sky glow from city lights even when the moon is below the horizon [30]. The nocturnal foraging behavior of *A*. *dorsata* might therefore be an example of positive light effects on activity, where the light available from the moon or artificial lights stimulate activity at a time the species would not otherwise be active [31]. Regardless, this ability to be active during bright daylight hours as well as dim nighttime hours is impressive, as light intensity decreases by a factor of one million between a sunny day to a full moon night and is therefore even lower on nights when the moon is not full [32].

The ability of *A*. *dorsata* to fly during the night and during the day is all the more intriguing given that while all three closely related species belonging to the *A*. *dorsata* complex (*A*. *dorsata dorsata*, *A*. *dorsata binghami* (Sulawesi), and *A*. *dorsata breviligula* (Philippines)) can fly during the night, the even larger Himalayan giant honey bee *A*. *laboriosa* cannot, though as mentioned this is likely due in part to colder nighttime temperatures in its range [19–22, 24]. Indeed, all other species in the genus *Apis*, with the possible exception of the African bee *A*. *mellifera adansonii* [33], are strictly limited to diurnal activity [26]. Given that body size and eye morphology alone cannot explain their nocturnal activity, what is it about the biology of *A*. *dorsata* that has led to the evolution of this ability? This question is challenging to answer because we lack basic information about the importance of nocturnal behavior in the foraging ecology of this species, and the environmental conditions that limit the ability to forage actively at night.

Regardless of the underlying mechanisms that permit crepuscular and nocturnal activity, it is likely that *A*. *dorsata* benefits from foraging in low light conditions. Nocturnal foraging by animals is often associated with reduced predation and reduced competition [26, 34–36]. There are also potential thermoregulatory advantages for night-active animals in warm locations such as the tropics, as the energy needed to reduce the risk of overheating is decreased [23, 37]. However, *A*. *dorsata* appears to have relatively low metabolic heat output in flight, so we would expect it to be disadvantageous at cooler nighttime temperatures [20, 29]. A particularly significant advantage of nocturnal activity for *A*. *dorsata* may be related to food gathering: by extending their diurnal activity into the night, they could prolong their ability to exploit flower species that offer pollen and nectar resources near sunset and sunrise, and gain access to an entirely new set of night-blooming floral resources [11, 38–40].

Here we present the first systematic investigation into the nocturnal behavior of *A*. *dorsata*. Over the course of eight months, we observed colonies of *A*. *dorsata* in Bangalore, India to examine how their diurnal and nocturnal behavior changed over the course of multiple seasons and lunar cycles. We paid particular attention to correlations between nocturnal activity and light availability, as well as between nocturnal activity and temperature, while also relating their behavior to expected resource availability in the surrounding environment. Our data reveal a rich picture of the environmental factors that influence this behavior.

We conducted our studies at a scientific research institute on the outskirts of a large city. The advantage of this study site was that we could work with colonies nesting on buildings, and thus could monitor foraging from multiple colonies at a close distance and over an extended period of time. The disadvantage of this site was that light pollution, in the form of “sky glow”, was present, and this may have enabled bees to forage at times they would not have been able to in areas without light pollution [30]. This limits our ability to fully generalize our conclusions to environments without light pollution. However, *A*. *dorsata* is widespread and successful in urban cities [41, 42], and so our observations are relevant for understanding the natural biology of free-living colonies. Furthermore, our systematic study can provide a baseline that could facilitate future research on the unique nocturnal behavior of *A*. *dorsata* in a wider range of environments.

## Materials and methods

### Study location

This study was conducted on the campus of the National Centre for Biological Sciences–Tata Institute of Fundamental Research (NCBS) in Bangalore, Karnataka, India, from October 2018 through May 2019. NCBS is located in a primarily residential district in the northern part of the city of Bangalore, adjacent to the University for Agricultural Sciences–Bangalore (GKVK). NCBS has extensive ornamental vegetation on its campus and is surrounded by fields of agricultural vegetation on the GKVK campus, as well as by residential tracts offering ornamental plants [30]. Because it is located on the outskirts of the urban city of Bangalore, NCBS is subject to light pollution.

### *Apis dorsata* biology

*Apis dorsata* can be found through southern Asia [43, 44]. It is one of the largest honey bee species, with a worker mass of ~120 mg when unloaded [45]. Like all species in its genus, *A*. *dorsata* is eusocial, with perennial colonies that reproduce by colony fission. Each colony consists of a single queen, a large number of workers, and a substantial but variable number of males (drones) during the swarming season. As foragers, the workers are ecological generalists and exploit a wide variety of floral species for nectar and pollen [46, 47]. Recruitment to food relies upon the dance language, similar to that of the Western honey bee, *A*. *mellifera* [48]. Colonies have up to 50,000 individuals, and nest in the open on a large (≈1m diameter) sheet of comb attached to an overhanging structure such as a tree branch, rock cliff face, or building ledge. Worker bees protect the colony by forming a protective curtain across the entire surface of the comb; foraging bees land on, depart from, and dance on this protective curtain [49–51]. Colonies of *A*. *dorsata* can be found in forests, agricultural areas, and urban areas, and often form aggregations of 10 or more colonies in a single location. *Apis dorsata* is a migratory species, traveling up to 200 km through a series of shorter steps to follow currently available resources [52–54]. *A*. *dorsata* colonies tend to be found within Bangalore during the dry season (which lasts from October to May), migrating into Bangalore in November and December and then migrating out of Bangalore in May and June before the start of monsoon season (which lasts from June to September) [41].

### Collection of *A*. *dorsata* colonies

**Fig 1:**
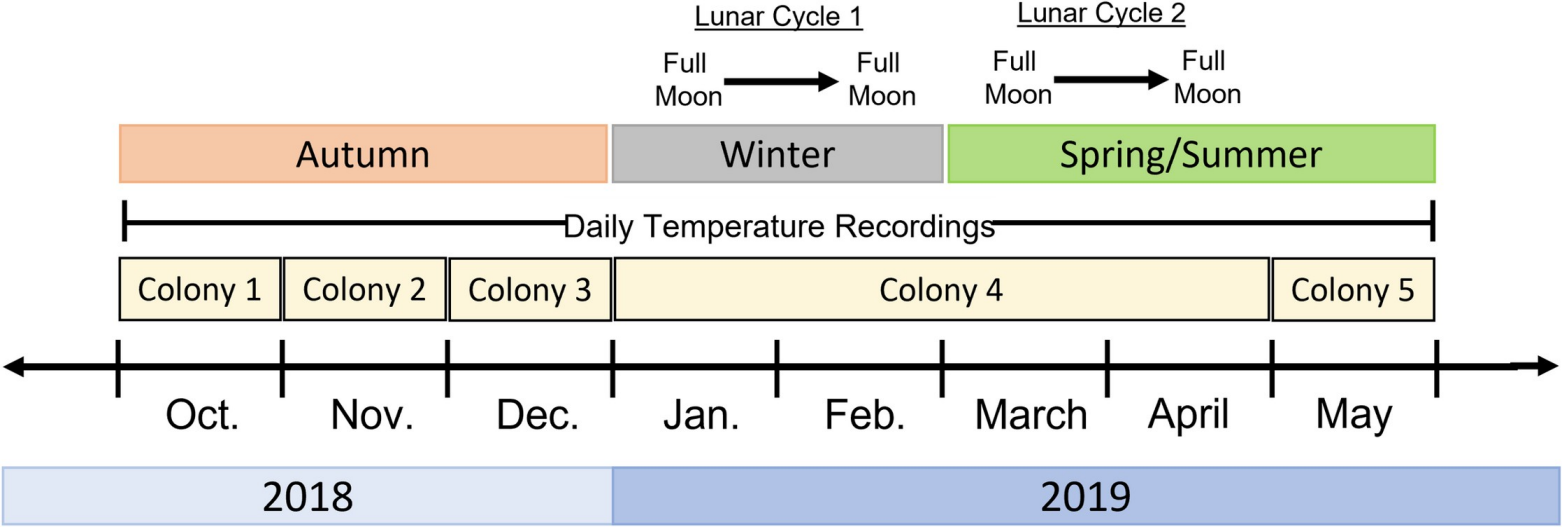
Timeline of data collection by month. We recorded nocturnal activity from 5 nights before to 5 nights after the full moon of every month except October and November, where we recorded nocturnal activity from the full moon to 5 nights after the full moon due to delays in obtaining *A*. *dorsata* colonies. The colony observed each month is shown, as is the season to which each month belongs. We obtained minimum temperatures for every observation day through an online resource (https://www.wunderground.com/history/daily/in/bangalore/VOBL). In addition to the observations focused on the full moon, we also observed Colony 4 for two complete lunar cycles, indicated by the text above; illumination readings were collected every observation night during the January to February lunar cycle (lunar cycle 1).

Five colonies of *A*. *dorsata* were observed over the course of this study (Fig 1). In October, November, and December, we collected one colony per month from the Bhartiya City apartment complex in Bangalore, which was located approximately 10 km from the NCBS campus. Colonies were collected with the approval of the apartment complex; no other permits or approvals were necessary to collect and observe *A*. *dorsata* colonies. We had to find and transport colonies from this apartment complex to the NCBS campus as no colonies were naturally occurring on the NCBS campus during these months. Colonies were found nesting under the overhanging balconies of the apartment buildings. To collect a colony, we followed the procedure outlined in [55, 56], where a bamboo stem that had been split down its length is used as a clamp to carry the comb after cutting it from the balcony. Care was taken to minimize the disturbance to the collected colonies. After collecting the colony, we transported it by truck to the NCBS campus and installed it in a large open-faced box on the roof of a building about 1m off the ground. Colonies were allowed to settle for at least 12 hours before observations were started. New colonies had to be collected every month as the transplanted colonies generally absconded (i.e., abandoned their nest) two to three weeks after being moved to the NCBS campus (minimum: 1 week, maximum: 3 weeks).

During January through May, we used *A*. *dorsata* colonies that were naturally nesting on buildings on the NCBS campus in this study. One colony was observed from January through April. After this colony migrated away from the NCBS campus, a second colony was observed during May.

### Monthly observations of nocturnal activity

We used a video camcorder (Panasonic 4K Ultra HD Model VX981K) placed 0.75m away from the colony to record the nocturnal activity of *A*. *dorsata* colonies on nights surrounding the full moons of the months of October through May (Fig 1). At this distance, we were clearly able to observe individual bees on the recordings, allowing for accurate observations of bee arrivals and dances (S1 and S2 Videos). Using the infrared setting, we recorded the colonies from shortly before sunset (generally around 1815 hours IST) to shortly after sunrise (generally around 0630 hours IST). As much as possible, we recorded the nocturnal activity of each *A*. *dorsata* colony from five nights before the full moon to five nights after the full moon. It was not always possible to record every night in this period every month. For example, during the months of October and November when the number of *A*. *dorsata* colonies present in Bangalore was low (because migrating colonies had not returned to the region), we were unable to collect *A*. *dorsata* colonies until the day of the full moon, and so colonies were only recorded from the night of the full moon onward for those months. Also, the occurrence of rain at night occasionally prevented us from recording nocturnal activity, though this was rare. Monthly observations were categorized into seasonal categories; October through December comprised “autumn”, January through February comprised “winter”, and March through May comprised “spring/summer” based on the suggestions of local Bangalore residents (Fig 1).

### Observations of diel activity patterns over lunar cycles

We recorded the nocturnal and diurnal activity of one *A*. *dorsata* colony for two complete lunar cycles: January 21 to February 19, 2019 and March 21 to April 19, 2019 (Fig 1). For each cycle, we began filming on the night of the first full moon (January 21 and March 21, respectively) and recorded for 24 hours until sunset on the following day. These 24-hour recordings were repeated every four to five days, such that we were able to record behavior on the night of every major moon phase in the lunar cycle (full, third-quarter, new, first-quarter, full) as well as one day in between each major phase.

During the January–February lunar cycle, we also took light measurements during every night when we recorded nocturnal activity. We used a Hagnar Universal Photometer/Radiometer S4 to record the illumination of the environment every hour, starting at sunset and continuing until sunrise. The sensor was on the roof above the studied *A*. *dorsata* colony and was oriented parallel to the ground and aiming skyward; a reading was recorded when the illumination value displayed was stable for 30 seconds.

### Video analysis

After recording, we analyzed videos to quantify the activity of *A*. *dorsata*. For all time periods when activity was recorded, we conducted a ten-minute activity census every half hour, counting the number of arriving bees and the number of waggle dances occurring during the census period. The times of sunset, sunrise, moonset, moonrise, and twilight (including the end of astronomical twilight and the beginning of astronomical dawn) were identified using the website https://www.timeanddate.com/moon/india/bengaluru. The end of astronomical twilight and beginning of astronomical dawn mark the beginning and ending of night, respectively, as light from the sun is not available in the sky and only the moon, stars, and artificial lights provide illumination. Based upon these astronomical references, activity occurring between sunrise and sunset was considered “diurnal activity”; activity occurring from sunset to the end of astronomical twilight or from the beginning of astronomical dawn to sunrise was considered “twilight activity”; and activity occurring between the end of astronomical twilight and the beginning of astronomical dawn was considered “night activity”. Daily temperature data from the Kempegowda International Airport Weather Station in Bangalore, India (14 km from the NCBS campus) was accessed and recorded from the Weather Underground website (https://www.wunderground.com/history/daily/in/bangalore/VOBL).

### Statistical analyses

We conducted exploratory data analyses in RStudio version 4.0.2 [57]. As a check of our assumption that bee arrivals reflect ongoing foraging during all portions of the diel cycle (hereafter referred to only as ‘diel time’), we ran Spearman correlation analyses on the number of arrivals and number of dances occurring in each 10-minute census period throughout each diel time (day, twilight, night). Our outcome variable of interest was the number of bees arriving back to the nest during the census period—hereafter referred to as “arrival rate”. This measure consisted of count data and was found to be over dispersed, so we used a negative binomial distribution in all analyses. Using our entire dataset, we fitted a generalized linear mixed-effect model to determine the effects on arrival rate of season (autumn, winter, spring/summer), minimum temperature, diel time (day, twilight, night), and the interaction between season and diel time, including the random factor of colony ID to account for differences between colonies (‘glmmTMB’ function [58]). We then subsetted the data to create datasets containing only those measurements collected for lunar cycle or illumination analyses; this subsection therefore contained only part of the data collected during the winter and spring/summer seasons, and no data collected during autumn. Separate generalized linear models were fitted to quantify (1) the effect of the lunar cycle and (2) the effect of environmental illumination on bee arrival rates (‘glmmTMB’ function [58]). The model for lunar cycle included the fixed factors of moon phase (first quarter, full moon, waning gibbous, waning crescent, new moon, third quarter, waxing crescent, waxing gibbous), lunar cycle ID (January, March), diel time (day, twilight, night), the interaction between diel time and lunar cycle ID, and the interaction between diel time and moon phase. The model for illumination included the fixed factors of illumination, time since sunset, and census time. Colony ID was not considered in the models for lunar cycle or illumination, as data were collected from only one colony during these periods. We built models using a forward approach, beginning with null models and adding complexity through the addition of fixed and random factors. We selected final models using a holistic approach based on their AIC values, R^2^ values, and performance during model diagnostic tests that checked for outliers, dispersion, and deviation (‘DHARMa’ package [59]). Final models selected were those with the lowest AIC, best model diagnostics, and highest R^2^ values. Descriptions of all models tested for each response variable, as well as performance in model selection criteria, can be found in Tables A, B, and D in S1 File. After selecting the final model, we analyzed the models using ANOVA, and analyzed significant factors using post hoc tests with a Tukey correction. Means are reported as the estimated marginal means plus or minus standard error; estimated marginal means are the means for each factor given by the final models chosen.

## Results

### General description of patterns of activity

**Fig 2:**
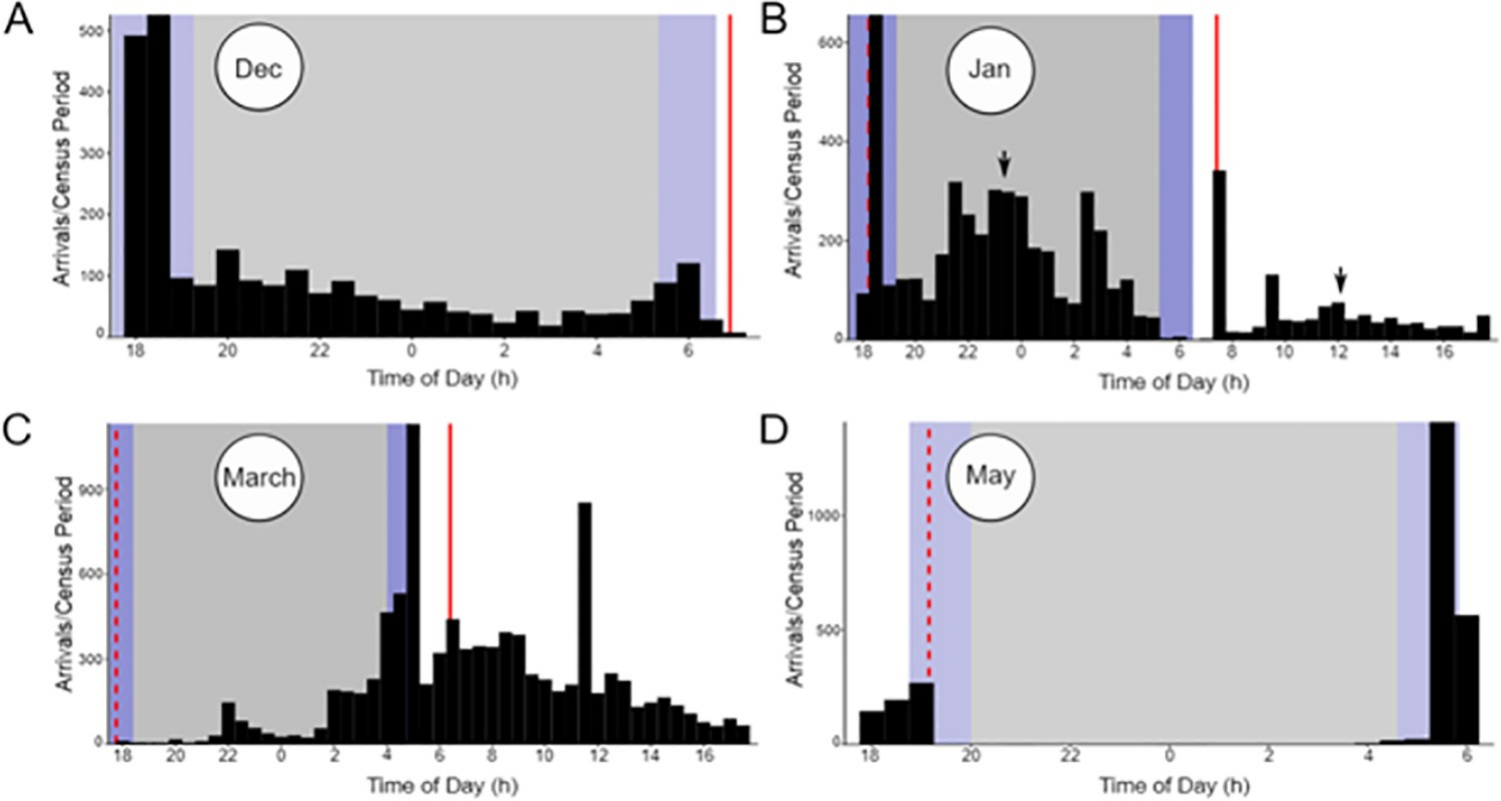
Daily foraging activity patterns of *A*. *dorsata*. Daily foraging activity on the days of the (A) December full moon, (B) January full moon, (C) March full moon, and (D) May full moon are shown as representative examples of *A*. *dorsata* daily activity patterns. Purple shaded regions indicate twilight, gray shaded regions indicate night, and white indicates daytime. The red dotted line indicates moonrise, while the solid red line indicates moonset. Arrows on the January full moon panel (B) indicate times corresponding to the sample videos provided in the supplementary materials (S1 and S2 Videos). Figures depicting the daily foraging activity for every day of the January–February and March–April lunar cycles can be found in S1 Fig. Data were collected from Colony 3 in December, Colony 4 in January and March, and Colony 5 in May.

We found that *A*. *dorsata* colonies exhibited peak nocturnal activity in the hours before and just after sunrise (generally 0400–0600 hours) and before and just after sunset (1700–1900 hours; Fig 2). While we analyzed activity during both astronomical twilight periods (a smaller portion of the time ranges just given) in our primary analyses, a supplemental analysis comparing activity during dawn versus dusk twilight showed that over the course of the study total activity did not differ between these time periods, though the proportion of activity that occurred during dawn versus during dusk did differ depending on the season (S2 File). When we observed activity across the lunar cycle, we further saw that activity during dawn and dusk combined generally represented the peak activity throughout the entire 24-hour day, at least in the colony studied at that time (Colony 4; Fig 2B, 2C, S1 Fig). Activity tended to be higher during the day when activity at night was low (such as during the new moon). Most bees that performed a recruitment dance returned to the hive with pollen, particularly at night (X±SE: day = 52.5%±2.2%, twilight = 86.3%±3.1%, night = 92.4%±1.7%). A surprising observation was that nocturnal activity was sometimes seen in the hours after astronomical twilight ended but before the moon had risen. On the nights of the January waning gibbous and third quarter moons in particular high activity was seen during this period. See S1 Fig for graphs showing daily activity patterns for every day recorded during the January to February and March to April lunar cycles.

**Fig 3:**
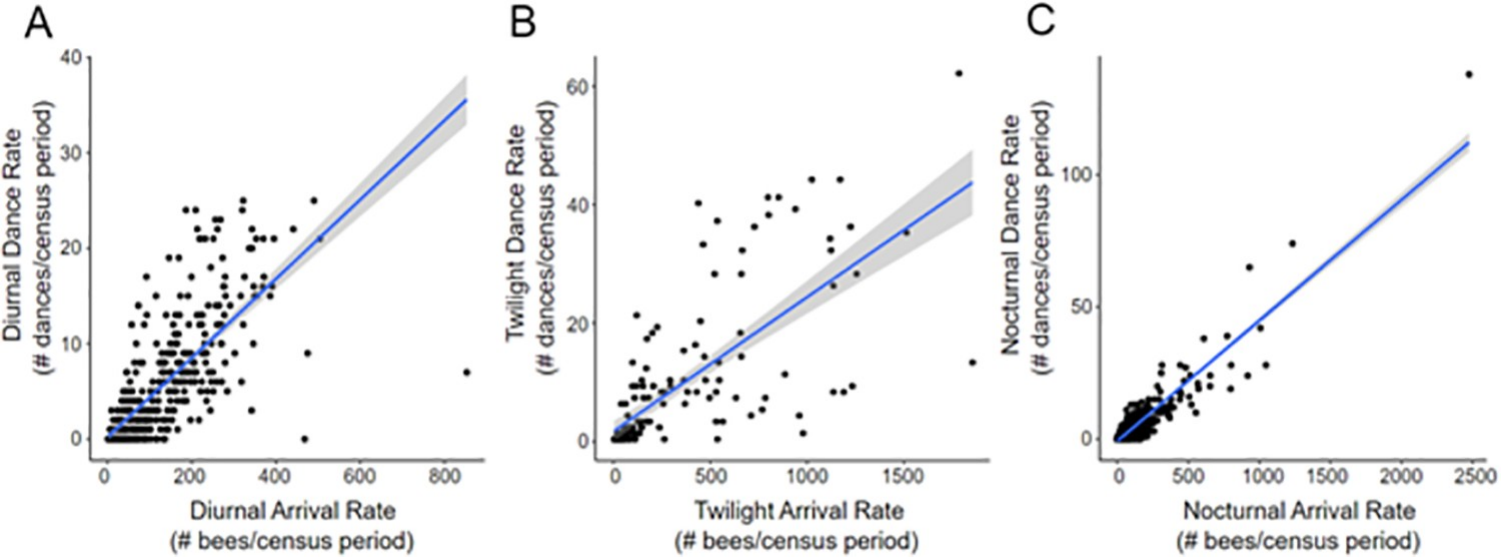
Relationship between bee arrival rates and dance rates. Regressions of number of bee arrivals against number of bee dances per census period are shown for (a) day time periods, (b) twilight time periods (both morning and evening), and (c) night time periods. Strong correlations were found between arrival and dance rates for all diel times (*r*_*s*_ > 0.82 for all). Data from all five *A*. *dorsata* colonies are included in this analysis.

A key assumption of our study is that bees seen arriving at the nest are returning from foraging flights (as opposed to learning flights or defecation flights). In support of this assumption, we observed strong positive correlations, at all parts of the diel cycle, between the number of bees arriving and the number of recruitment dances during the same census period (day: Spearman *S* = 2461556, *r*_*s*_ = 0.82, *P* < 2.2e-16; twilight: *S* = 94819, *r*_*s*_ = 0.84; *P* < 2.2e-16; night: *S* = 5921424, *r*_*s*_ = 0.82, *P* < 2.2e-16; Fig 3). The correlations seen at night are especially important: since dances are performed only immediately following a forager’s return from a successful foraging trip observing strong correlations at night confirm that counts of nocturnal arrivals can be taken as a measure of nocturnal foraging rates. Because these correlations include all data collected over the course of the observations on our five study colonies, we are confident that the correlations are robust.

### Nighttime activity in relation to illumination

**Fig 4:**
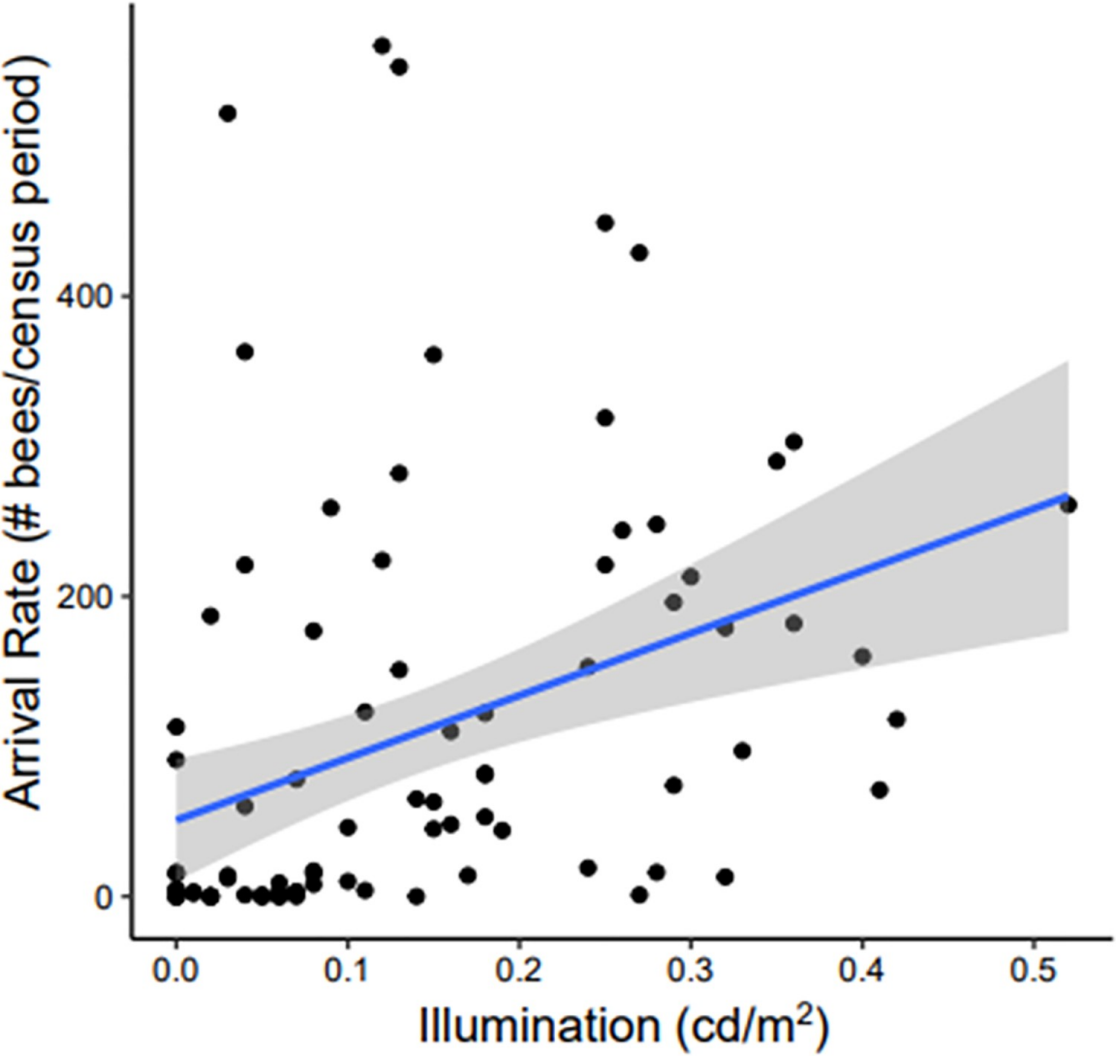
Relationship between nighttime illumination and bee arrival rates. A regression between environmental illumination at night and number of bee arrivals to the nest per census period is shown. Illumination had a significant positive effect on arrivals, such that as illumination increased arrivals also increased (*r*_*s*_ = 0.56, *P* = 0.0084). Arrivals were seen even when illumination was recorded as 0 cd/m^2^ at the limit of the photometer’s sensitivity. Data shown here were collected exclusively from *A*. *dorsata* Colony 4.

During the night, illumination had a significant positive effect on bee arrivals ($F_{86}^{1}$ = 6.94, *P* = 0.0084): as illumination increased the arrival rate also increased (*r*_*s*_ = 0.56; Fig 4). Surprisingly, we observed arrivals even when illumination was recorded as 0 cd/m^2^ (i.e., at the limit of the photometer’s sensitivity). The amount of time that had passed since sunset did not have a significant effect on the arrival rate, nor did the time at which we censused arrivals. We were only able to record illumination during the month of January, and so this correlation between illumination and arrivals derives from the observations of Colony 4 alone.

### Activity patterns over the lunar cycle

**Fig 5:**
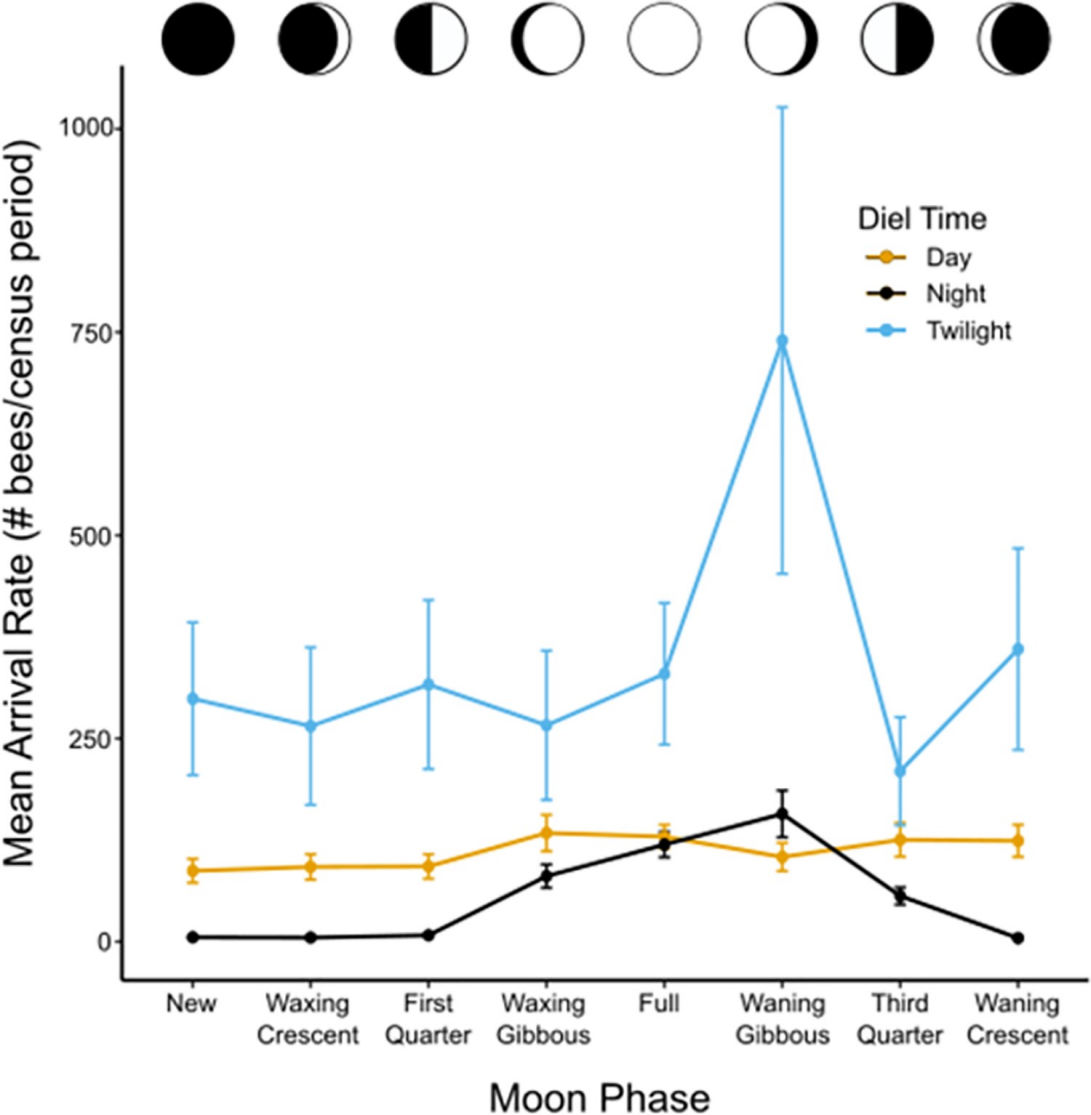
Estimated marginal mean arrival rates across moon phases. Estimated marginal mean arrivals rates during different diel times are shown across moon phases. Activity was consistently the highest and equal during the day and twilight (all *P* > 0.05) except during the waning gibbous and waning crescent moons (*P* < 0.05) where more activity was seen during twilight time periods. Nocturnal activity was generally low compared to activity the rest of the day (*P* < 0.05), except the moon phases including and between the waxing gibbous and third quarter moons, where it was equal to activity seen during the day (*P* > 0.05). Error bars represent 95% confidence intervals. Data shown here stem exclusively from observations of *A*. *dorsata* Colony 4, and include only data collected during the lunar cycle observation periods from January 21, 2019 –February 19, 2019 and March 21, 2019 –April 19, 2019.

When examining the effects of moon phase on bee arrival rates, we found that arrival rates varied greatly depending on diel time ($F_{807}^{2}$ = 437.58, *P* < 2e-16) and moon phase ($F_{807}^{7}$ = 291.9286, *P* < 2e-16), and that the activity seen during a given diel time varied depending on the moon phase (Diel Time*Moon Phase: $F_{807}^{14}$ = 317.0116, *P* < 2e-16; Fig 5). In particular, all bee arrival rates during the day (all *P* > 0.9) and during twilight (all *P* > 0.5) were constant across the moon phases. Arrivals rates during the day and during twilight on a given moon phase generally did not differ (*P* > 0.05), except for on the night of the waning gibbous moon, where twilight arrival rates were higher than daytime arrival rates (*P* < 0.05). In contrast, arrival rates at night were highly dependent on proximity to the full moon (Fig 5), being high and close or equal to the daytime arrival rates within a week of the full moon but extremely low and close to zero near the new moon (see Table C in S1 File for all estimated marginal means). Arrival rates at night were often lower than arrival rates during twilight (all *P* < 0.05) but did match twilight arrival rates on the nights of the full, waxing gibbous, waning gibbous, and the third quarter moons (all *P* > 0.05).

**Fig 6:**
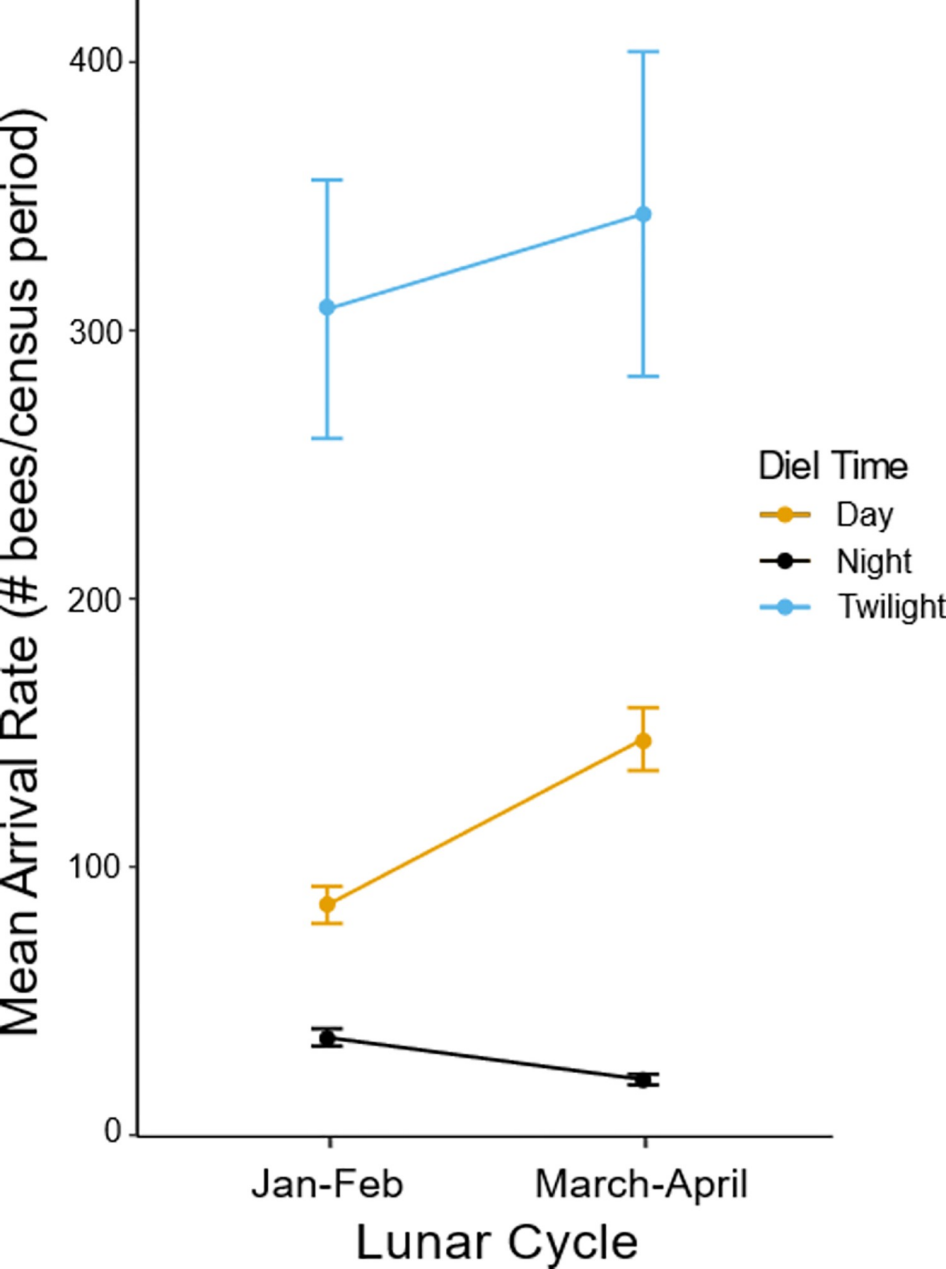
Estimated marginal mean arrival rates in each lunar cycle. Estimated marginal mean arrival rates during each diel time over both lunar cycles are shown. In both lunar cycles the highest number of arrivals were seen during twilight (*P* < 0.001), and the least number of arrivals were seen during the night (*P* < 0.001). An interaction was seen where nocturnal activity decreased but diurnal activity increased from the January lunar cycle to the March lunar cycle (*P* < 0.001). Error bars represent 95% confidence intervals. Data shown here stem exclusively from observations of *A*. *dorsata* Colony 4, and include only data collected during the lunar cycle observation periods from January 21, 2019 –February 19, 2019 and March 21, 2019 –April 19, 2019.

Consistent with these patterns, variation in bee arrival rates at a given diel time was dependent on the lunar cycle (Diel Time*Lunar Cycle: $F_{807}^{2}$ = 36.77, *P* = 1.04e-8), though there was not a difference in total activity between lunar cycles ($F_{807}^{1}$ = 1.2421, *P* = 0.27). Arrival rates during the night were higher in the January lunar cycle than the March lunar cycle (*P* < 0.001; X±SE: March = 2.85±0.096, January = 3.41±0.092; Fig 6), but arrival rates during the day were higher in the March lunar cycle than the January lunar cycle (*P* < 0.001; X±SE: March = 4.98±0.08; January = 4.43±0.082). Arrival rates during twilight did not differ between lunar cycles (*P* = 0.99), but twilight had the highest rates of activity within both lunar cycles (*P* < 0.001; X±SE: March = 5.84±0.018, January = 5.73±0.16). Diurnal arrival rates were the next highest in both lunar cycles, with nocturnal activity the lowest in both cycles (*P* < 0.001 all). Data for both moon phase and lunar cycle analyses stem exclusively from observations of *A*. *dorsata* Colony 4 during the lunar cycle observation periods from January 21, 2019 –February 19, 2019 and March 21, 2019 –April 19, 2019.

### Activity patterns based on season and temperature

**Fig 7:**
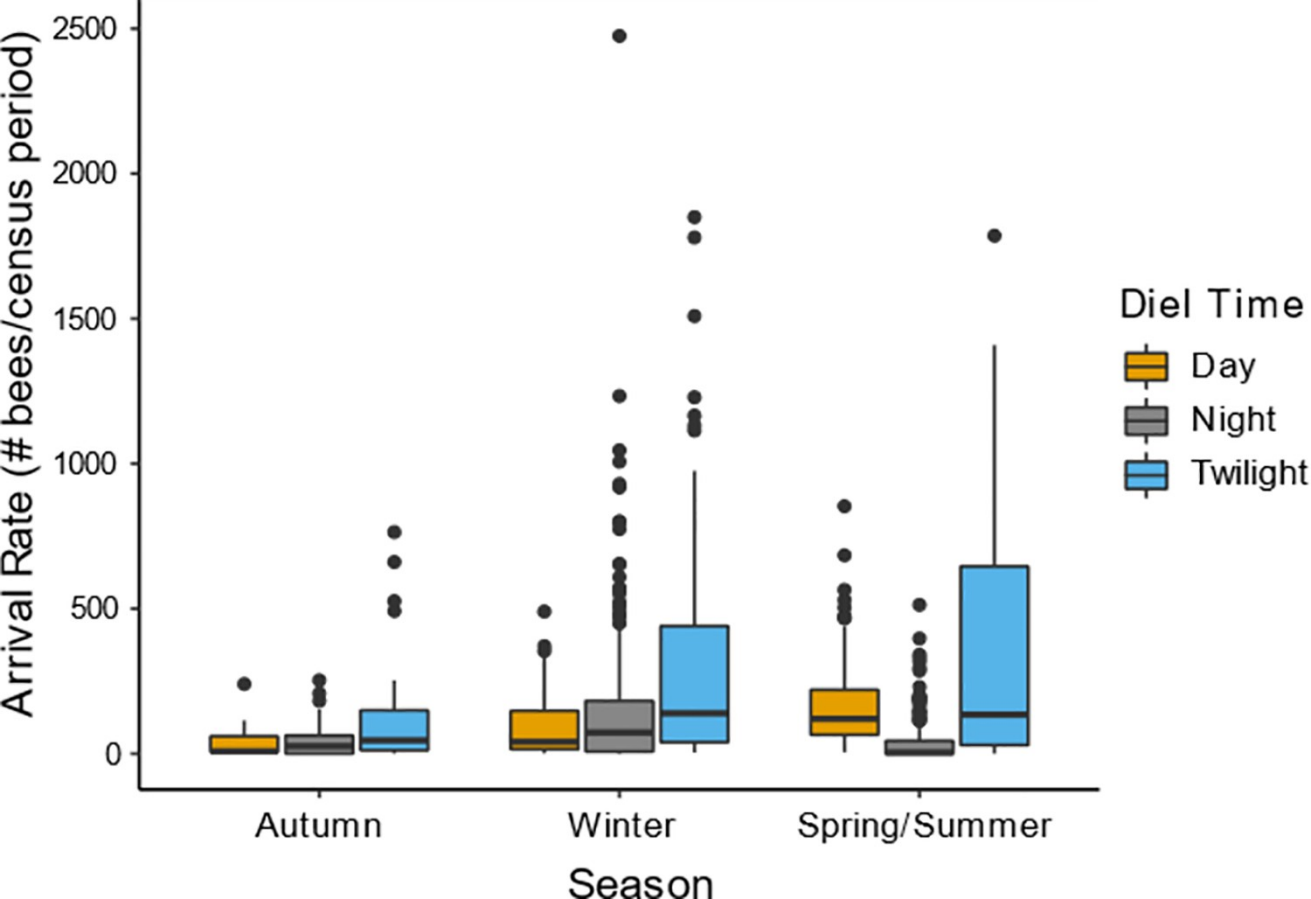
Arrival rates across seasons. Boxplots showing arrival rates during each diel time across seasons are shown. Nocturnal arrival rates were higher and diurnal arrival rates were lower in winter compared to spring/summer (*P* < 0.05), but did not differ between autumn and spring/summer or autumn and winter (*P* > 0.05). In both winter and spring/summer arrival rates were significantly different between every diel time, with twilight having the highest rates. Arrival rates were higher at night than during the day in winter (*P* < 0.05), which was not seen in any other season. Arrival rates during twilight was also higher than the nocturnal arrival rate in autumn (*P* < 0.05), but the arrival rates during twilight did not significantly differ between seasons (*P* > 0.6). The significance of all pairwise differences can be found in Table E in S1 File. Data shown here includes all observations from all five focal colonies studied over the course of the investigation.

When we examined all data collected from the five colonies observed over the course of the study, bee activity varied significantly with diel time ($F_{1237}^{2}$ = 151.22, *P* < 2e-16), and the activity seen at a given diel time differed among seasons (Diel Time*Season: $F_{1237}^{4}$ = 127.37, *P* < 2e-16; Fig 7). Contributing to this interaction, arrival rates during the night were higher in winter than in spring/summer (*P* < 0.001; X±SE: winter night = 4.58±0.63; spring/summer night = 3.30±0.63), but arrival rates during the day were lower in winter than in spring/summer (*P* < 0.001; winter day = 4.08±0.63; spring/summer day = 4.87±0.62). During the winter season, in fact, nocturnal arrival rates were higher than diurnal rates (*P <* 0.05; note that the winter season includes data from the lunar cycle observation periods discussed in the previous section as well as additional observations before the January full moon and after the February full moon). During autumn, arrivals rates were the same regardless of diel time (all *P* > 0.05), except for higher arrival rates during twilight than during the night (*P* < 0.05). In both winter and spring/summer though, there was more activity during twilight than during the day or the night (all *P* < 0.05).

**Fig 8:**
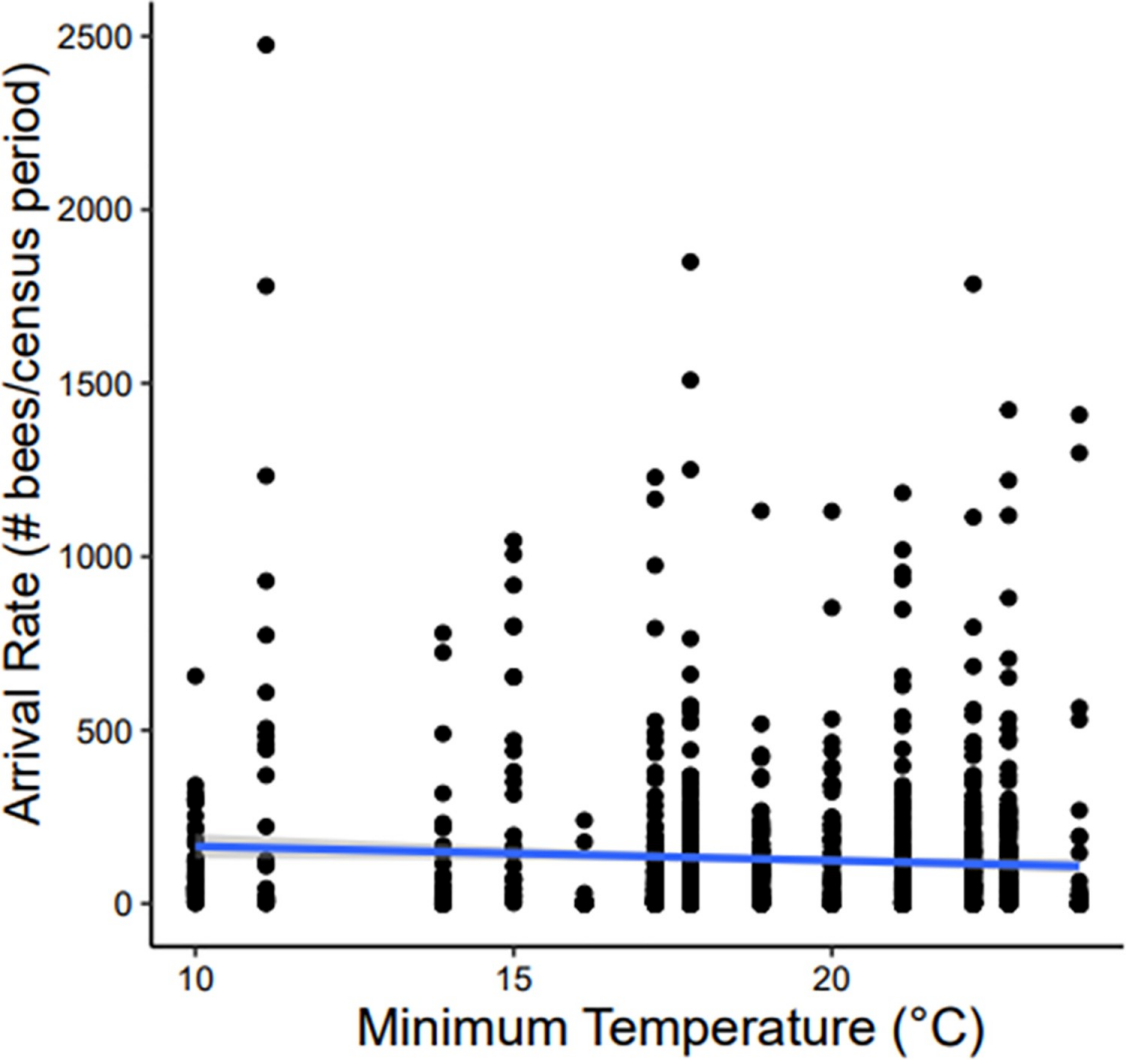
Relationship between minimum temperature and arrival rate. A regression between minimum daily temperature and bee arrival rates throughout the day are shown. There was no relationship between minimum temperature and bee arrivals, with high activity seen even during the coldest nights in winter (*P* > 0.05). Data shown here includes all observations from all five focal colonies studied over the course of the investigation.

Minimum daily temperature over the course of the study ranged from 10° - 24˚ Celsius. We found no relationship between minimum temperature and bee arrival rate in this experiment ($F_{1237}^{1}$ = 2.04, *P =* 0.15; Fig 8).

## Discussion

In this study, we provide the first systematic investigation into the nocturnal foraging behavior of the giant honey bee, *A*. *dorsata*, across multiple seasons. We observed the activity of *A*. *dorsata* over a period of eight months to examine environmental correlates of their diurnal, crepuscular, and nocturnal activity. Changes in activity during these time periods were dependent on the season, moon phase, and illumination of the environment, but did not seem to be influenced by temperature. Our results indicate that, at least in urban environments, foraging during crepuscular and nighttime periods make up a substantial portion of a colony’s daily foraging activity, and that this low-light activity was more important in some seasons than in others.

Activity during crepuscular and nighttime periods could be the result of bees actively foraging for resources or simply due to bees returning to their nest at the end of their day’s work. In support of the first interpretation, arrival rates and dance rates were highly and similarly correlated during the day, twilight, and night in this study (Fig 3), providing strong evidence that bees are actively foraging during the crepuscular and nighttime hours. In addition, the majority of bees dancing during those nocturnal hours returned with pollen, further supporting our underlying assumption that arrival rate is an accurate metric for assessing foraging effort. Arrival rates and dance rates were not perfectly correlated, presumably either because bees observed arriving were foragers that returned to the nest and did not dance or because they were away from the nest for some other reason. An additional source of noise could be due to the additional flights *A*. *dorsata* colonies perform during certain times of the day. Drones frequently perform mass mating flights at sunset [60–63], and workers often perform mass orientation and defecation flights early in the morning and during the hottest parts of the day [63–64]. However, mass flights are highly distinctive in open-nesting species and can be characterized by the formation of wide gaps in the bee curtain, chains of bees, and increased general activity [65, 66]. We did not observe such signs of mass flights during any of our census periods, nor did we observe massive drone flights. Meanwhile, the strong and consistent correlations between arrival and dance rates suggest that most arrivals are workers returning to the nest at the completion of foraging flights.

These findings strengthen previous claims that *A*. *dorsata* actively forages at night when the moon is bright [11, 16, 17, 40]. They also provide a starting point for addition studies investigating the proportion of the foraging workforce that forages at night, whether the same individuals forage during different diel times, and whether the same resources are exploited during different diel times (as has been seen in resource overlap between diurnal and nocturnal carpenter bees [39]). While we cannot currently interpret the waggle dances observed in this study because the reference compass system for nocturnal dances is unknown [16, 30], we are in the process of analyzing the dances to determine the reference. With that information, future studies could also examine and compare the number of resources being exploited during different diel times and the locations of resources being exploited.

*Apis dorsata* colonies exhibited consistent peaks in activity at sunrise and sunset across the study period (Fig 2). The high activity seen from the start of astronomical dawn to sunrise is of particular note as it generally began to increase rapidly hours before and declined soon after sunrise. This early start to foraging is likely to provide a competitive advantage [26, 34–36], allowing *A*. *dorsata* foragers to exploit resources before other pollinators that need the light of the sun to forage [26]. The continuation of foraging throughout twilight and into the night is likely a similar extension of diurnal foraging, allowing *A*. *dorsata* foragers to continue exploiting profitable resources with minimal competition while taking advantage of new resources that become rewarding at night (e.g., some *Dipterocarpus* species [38], *Bridelia retusa*, *Randia dumetorum* [11], *Pterocarpus santalinus* [40]).

The amount of light available during the nocturnal hours was the primary determinant of nighttime foraging. While this partially supports the idea that nocturnal foraging is an extension of diurnal foraging, the low nocturnal activity seen during the waxing moon phases when the moon is up during the first half of the night (Fig 5, S1 Fig) suggests that nocturnal activity might not be solely an extension of diurnal foraging. However, the low activity during this time might alternatively be due simply to the resource base being depleted after diurnal exploitation. As illumination in the environment increased, the activity of bees during the night similarly increased (Fig 4). These results suggest that the nocturnal behavior of *A*. *dorsata* is an example of positive light effects on activity, or “masking”, whereby increased illumination stimulated activity outside of the endogenous circadian rhythm, as has been seen in other diurnal species [4, 67]. This illumination was primarily, but not solely, provided by the moon. with high nocturnal activity seen only within the week before to the week after a full moon, the period when the moon was at least half full (Fig 5). While light pollution at our study site provided some additional illumination, it did not completely change the behavior of foraging workers, as their periods of high nocturnal activity correspond with and support previous reports of *A*. *dorsata’s* nocturnal activity [11, 16, 30].

Interestingly, nocturnal activity was often higher during the waning phase of the lunar cycle as compared to when the moon was waxing (Figs 2 and 5), even though the total amount of nocturnal illumination should be the same on these nights, all else being equal. One explanation for this may be that foraging during the waning phase would benefit from having increased information and learning after multiple nights of foraging activity. When the moon was waxing, by contrast, nocturnal foragers would be in the process of learning when and where they could forage at night. Alternatively, the increased activity seen during the waning lunar phases could be a result of the waning moon still being high in the sky in the hours before sunrise; this would enable *A*. *dorsata* foragers to get a competitive edge in exploiting morning-blooming flowers [33, 68]. During the waxing phase of the moon, by contrast, when the moon is high in the sky at sunset, the ability to fly after sunset would offer a weaker competitive advantage in exploiting flowers that had already been heavily exploited by diurnal nectarivores. Finally, it is possible that this difference in nocturnal activity during the waxing vs waning phases of the lunar cycle could be due to properties of the bees’ circadian clock. European honey bees (*A*. *mellifera*) are reported to have rhythmic circadian patterns of activity that are shorter than 24 hours (reports range from 21.8–23.5 hours [69]). Studies have shown that humans, who have a circadian pattern of activity that is longer than 24 hours, are more responsive to moonlight in the early night (such as when the moon is waxing) than during the later night and early morning [70]; it is possible that the opposite pattern is true for species with patterns of activity that are less than 24 hours. Though we currently have no information on the circadian activity rhythms of *A*. *dorsata*, if they have a shorter rhythmic activity pattern similar to *A*. *mellifera* then we might expect them to be more responsive to illumination in the latter half of the night and early morning as compared to the first half of the night. High activity during the waning lunar phases and in the hours before sunrise, then, would be unsurprising given the moon would still be high during the latter half of the night.

Though nocturnal activity was highly dependent on the illumination provided by the moon, bees were able to forage throughout twilight periods even without the moon’s illumination. Even when illumination was recorded to be 0 cd/m^2^ (i.e., at the lowest sensitivity limit of the photometer), bees were still actively foraging (Fig 4). The photometer we used was calibrated for human vision and may not have been sufficiently sensitive to ultraviolet wavelengths to which bees are sensitive [71]). Alternatively, the recording location might have underestimated the light available to foraging bees, as we only took light measurements near the nest. Given our study site was the NCBS campus in Bangalore, there were point sources of light throughout the campus that could have provided additional illumination our readings could not account for which might have provided bees with additional light. In any case, our observations during twilight on nights when the moon is not visible are striking evidence that bees can fly when there is even less light than what is provided by a half moon.

One possible reason why *A*. *dorsata* was able to forage so extensively during the twilight periods is due to the previously mentioned anthropogenic light in our study location. Because of anthropogenic “sky glow”, the nocturnal sky brightness in Bangalore is on average 20 to 40 times brighter than the natural nocturnal sky unpolluted by human light; this means that the nighttime sky has the same brightness as during nautical twilight and is therefore brighter than during astronomical twilight [72]. We found evidence that this urban sky glow was sufficient for nighttime foraging even when the moon is not up, as seen by the small amount of foraging activity after astronomical twilight and before moonrise on the nights of the January waning gibbous and third quarter moons (S1 Fig)

We found no evidence that nocturnal foraging on a given night leads to a subsequent change in diurnal foraging. Instead, colony activity during the day and during twilight remained relatively constant and equal regardless of the amount of nocturnal activity observed (Figs 5 and 6). Assuming that the same individual bees can forage both day and night, this consistent diurnal and crepuscular activity across the lunar cycle suggests that nocturnal activity is not due to changes in the bees’ circadian clock entrainment by increased moonlight, as daily activity patterns would be expected to shift in parallel with the moon phases in that case [3]. Instead, these results again support the idea that nocturnal activity is the result of positive light effects on activity by environmental illumination that allows bees to extend their temporal niche without shifting their circadian clock [2, 3, 7]. However, research in *A*. *mellifera* workers has shown that the times at which foraging workers are active can have a substantial influence on the cycling of clock gene expression [73]. Future work investigating investment in nocturnal activity in relation to clock gene expression within *A*. *dorsata* workers could clarify the mechanisms by which nocturnal activity occurs, namely if it is due solely to these positive direct light effects or if it is also due to changes in circadian clock entrainment.

Although arrival rates during nighttime were typically the lowest among the three time periods studied in each diel cycle, nocturnal arrival rates around the full moon were equal to those seen during the day (Fig 5), suggesting that nighttime foraging can contribute a large portion of a colony’s daily activity, energy expenditure, and resource collection. The high rates of activity seen across all diel times suggest that this species could be considered cathemeral (active during both the light and dark portions of the daily cycle), at least during the days of the lunar cycle when the moon is at least half full. However, given that the number of total colonies followed here was low and at times data were analyzed from only one colony, and given that urban sky glow could have influences our observations, additional studies should be conducted to determine how well our observations generalize to *A*. *dorsata* in other environments.

While ambient light availability in the environment seems to be necessary for nocturnal activity, the amount of activity seen during the day, twilight, or night also seems to vary with the seasons. Nocturnal activity was highest in the winter months (Fig 7) and particularly high in the winter months of January and February compared to the spring/summer months of March and April (Fig 6). This seasonal change in nighttime activity correlates with seasonal variations in resource availability, as many night blooming flower species are in peak season from December to March in southern India (e.g., the *Balsaminaceae* family [74]). As we observed colonies during the full moon period every month, light availability must not be the factor restricting bees’ nocturnal foraging during autumn or spring/summer; instead, it seems more likely that nocturnal foraging is responsive to the rewards currently available in the environment. Across all seasons, activity during twilight periods was consistently higher than activity during the day or the night (Fig 7), suggesting that resource availability during these diel times is high regardless of season. In contrast, the higher diurnal activity seen during autumn and spring/summer than during winter suggests that diurnal resource availability was high during these periods. The high activity during spring/summer was surprising because the months of April to June tend to have fewer native floral resources than other months in southern India [75, 76], though at least one tree species flowers during this period (*Peltophorum pterocarpum* [77]). However, as most of the resources located within the foraging range of these colonies are cultivated species in the NCBS gardens and botanical garden, the flowering phenology of these plant species might not match that of native forests in the same part of India. Instead, it is likely that floral resources are available throughout the year near the NCBS campus due to these ornamental plant species, limiting possible effects of seasonal resource availability on *A*. *dorsata’s* activity. In addition, resources are available throughout the year in India even without access to ornamental plants like the bees in our study had [75, 76], further supporting the possibility that resources were not limiting in any season. Another possible explanation for the high diurnal activity in spring/summer is that one of the colonies being studied was preparing to migrate, as it abandoned its hive in the beginning of May. Possibly then, the increased diurnal activity might represent both foraging efforts and scouting behavior of bees looking for new nest sites, though this is unlikely as pre-migratory dances are noticeably longer and more variable than foraging dances [78]. The seasonal availability of floral resources (diurnal or nocturnal) in urban cities such as Bangalore has not been systematically studied, or at least has not been published, but is of critical importance to understanding the foraging behavior of pollinator species such as *A*. *dorsata* that live in these habitats.

Although we expected to find an influence of temperature, ambient temperature was not a limiting factor for *A*. *dorsata’s* nocturnal foraging (Fig 8). During the 8-month study period, minimum daily temperature varied over a 14˚C range, and never dipped below 10˚C even at night during the colder winter months. Surprisingly, it was on some of the coldest nights in January when *A*. *dorsata* exhibited their highest nocturnal activity. While thoracic temperature in endothermic insects generally increases with body size, *A*. *dorsata* workers have a disproportionately low mass-specific metabolic rate which limits their ability to fly in cooler temperatures [20, 29]. However, while 10˚C is on the lower end of *A*. *dorsata’s* reported thermal tolerance, it is still within the normal bounds at which workers can maintain flight [29]. Thus, temperature did not limit flight at least in part because it simply did not get cold enough to impair the ability to maintain a high enough thoracic temperature to fly. We might have observed an effect of temperature had we performed this study at the northern limit of the range of *A*. *dorsata* (37.1°N in India), where the temperature can be much cooler than in Bangalore (12.97°N [43, 44].

Our study location at a research campus on the edge of the large urban city of Bangalore provided the unique ability to systematically study *A*. *dorsata* colonies long term. However, it also meant that the study location was both more light polluted and more sheltered from seasonal changes in resource availability than many of the habitats in which *A*. *dorsata* is found (e.g., forests). Because of those differences, some of our results cannot be generalized to all environments. Our findings that the nocturnal activity of *A*. *dorsata* is highly dependent on illumination and moon phase does match previous reports of their behavior [11, 16, 40]. However, the amount of activity seen during twilight periods and during periods of low illumination as measured by the photometer might have been boosted by the sky glow of Bangalore, as compared with what might be seen in more natural systems. Similarly, our conclusion that seasonal variation in activity across the diel time is influenced by seasonal changes in resources is likely to generalize to other urban and natural systems given the high seasonal variability in resource availability in India [75, 76], even if the urban population we studied might have been relatively buffered from resource fluctuations. To the extent that our observations are at least qualitatively generalizable to all systems in which *A*. *dorsata* is found, then our results provide a critical perspective on the nocturnal behavior of *A*. *dorsata* not only in light-polluted areas where *A*. *dorsata* is common [41, 42], but also to rural or forested environments to which these populations migrate. In addition, as the first systematic study of this behavior, our study provides a good starting point for future research into how *A*. *dorsata* forages at night and how their behavior differs depending on the environment.

Our results suggest that, at least in urban systems with light pollution, *A*. *dorsata* should not just be considered a diurnal species that can sometimes forage during twilight or at night, but a cathemeral species when illumination is sufficient to allow for positive direct light effects on activity. There seems to be a tendency toward “crepuscular” activity, but that may simply be a result of an abundance of flower resources combined with sufficient light to permit flight. By studying single colonies over an extended period of time, we provide an initial systematic investigation of the nocturnal foraging behavior of *A*. *dorsata* in urban systems, but more research is necessary to draw broad conclusions about the generality of these findings to other systems such as tropical forests without light pollution. We suggest research efforts also be exerted to gain a better understanding of the resource availability at night versus during the day and across seasons in the range of *A*. *dorsata* to further clarify the relationship between activity, illumination, resource availability, and season.

## Supporting information

S1 File Model selection tables from statistical analyses. This file contains model selection tables for (A) the effects of illumination; (B) the effects of moon phase, lunar cycle, and diel time; and (D) the effects of season and temperature on arrivals. Final models selected are highlighted in bold. It also contains (C) estimated marginal means for the interaction between moon phase and diel time for the model of effects of moon phase, lunar cycle, and diel time, as well as (E) a table showing pairwise differences in arrival rates during each diel time across the seasons.
(DOCX)

S2 File Dawn versus dusk supplemental analysis. This file contains the results of a supplemental analysis comparing activity during the dawn twilight period to activity during the dusk twilight period across seasons. (A) The ANOVA output for the statistical analysis and (B) a summary figure of the results are included.
(DOCX)

S1 Fig Daily foraging activity patterns of *A*. *dorsata*. Daily foraging activity on each day recorded during the January to February (left) and March to April (right) lunar cycles are shown. Purple shaded regions indicate astronomical twilight, gray shaded regions indicate night, and white indicates daytime. The red dotted line indicated moonrise, while the solid red line indicates moonset. Activity tended to peak during the twilight periods and was only seen at night during the full moons and the weeks before and after them.
(DOCX)

S1 Video Example of diurnal recording. A short, annotated clip of a video taken during the day on January 22, 2019 (start time 1215 h) is included to serve as a representative example of our diurnal video data. Pink circles during the video show examples of dancing *A*. *dorsata* foragers. Other dances occur during the video but are not marked. A longer unannotated video from the same day provides a larger variety of foragers landing and dancing and can be found at https://www.dropbox.com/s/tv3jcozp6km8p1l/22-1-19_1215_long.mov?dl=0.
(MP4)

S2 Video Example of nighttime recording. A short, annotated clip of a video taken during the night on January 21, 2019 (start time 2325 h) is included to serve as a representative example of our nocturnal video data. Three pink circles during the video show examples of dancing *A*. *dorsata* foragers; note all three dancers carry pollen. Other dances occur during the video but are not marked. A longer unannotated video from the same night provides a larger variety of foragers landing and dancing and can be found at https://www.dropbox.com/s/1p666dwrgx95knl/21_3_19_2325_long.mp4?dl=0.
(MP4)

S1 Data Analysis script. R analysis script. The script was used to analyze all data discussed in the manuscript. Data was analyzed in RStudio.
(R) Click here for additional data file.

S1 Datasheet Dorsata activity datasheet. This CSV file was used for the analyses discussed in the manuscript.
(CSV)

S2 Datasheet Moon phase only datasheet for graphs. This CSV file was used for creating the moon phase related graphs in this manuscript. The only differences from the dorsata activity datasheet are: it includes only the data collected during the moon phase observations and it includes an additional column for moon phase that allows the moon phases to be easily graphed in order of appearance instead of alphabetically by their name.
(CSV)
