# Supplementary material for: A hard day’s night: Patterns in the diurnal and nocturnal foraging behavior of Apis dorsata across lunar cycles and seasons
Source: PLoS One. 2021 Oct 22;16(10):e0258604. doi: 10.1371/journal.pone.0258604 (PMC8535376; doi:10.1371/journal.pone.0258604)
Supplement: S1 File — This file contains model selection tables for (A) the effects of illumination; (B) the effects of moon phase, lunar cycle, and diel time; and (D) the effects of season and temperature on arrivals. Final models selected are highlighted in bold. It also contains (C) estimated marginal means for the interaction between moon phase and diel time for the model of effects of moon phase, lunar cycle, and diel time, as well as (E) a table showing pairwise differences in arrival rates during each diel time across the seasons. (DOCX) [file pone.0258604.s001.docx]

**S1 Table A. Model selection for the effects of illumination, census time, and time since sunset on arrivals.** Final model selection is highlighted in bold.

| Model | AIC | R2 | Dispersion | Deviation | Outliers | Standardized Residuals |
| --- | --- | --- | --- | --- | --- | --- |
| (Null model) Arrivals ~ 1 |  | 0 | NS | NS | NS | Good |
| Arrivals ~ Illumination | **924.55** | **0.30** | **NS** | **NS** | **NS** | **Minor quantile deviations detected** |
| Arrivals ~ Illumination + CensusTime | 926.46 | 0.30 | NS | NS | NS | Major quantile deviations detected |
| Arrivals ~ Illumination + log(CensusTime) | 926.51 | 0.31 | NS | NS | NS | Major quantile deviations detected |
| Arrivals ~ Illumination + TimeSinceSunset | 926.40 | 0.30 | NS | NS | NS | Major quantile deviations detected |
| Arrivals ~ Illumination + log(TimeSinceSunset) | 926.55 | 0.31 | NS | NS | NS | Major quantile deviations detected |
| Arrivals ~ Illumination + CensusTime + TimeSinceSunset | 928.40 | 0.30 | NS | NS | NS | Minor quantile deviations detected |
| Arrivals ~ Illumination + CensusTime + log(TimeSinceSunset) | 928.49 | 0.31 | NS | NS | NS | Major quantile deviations detected |
| Arrivals ~ Illumination + log(CensusTime) + TimeSinceSunset | 927.94 | 0.33 | NS | NS | NS | Major quantile deviations detected |
| Arrivals ~ Illumination + log(CensusTime) + log(TimeSinceSunset) | 928.49 | 0.31 | NS | NS | NS | Major quantile deviations detected |

**S1 Table B. Model selection for the effects of moon phase, lunar cycle, diel time, and associated interactions on arrivals.** Final model selected is highlight in bold.

| Model | AIC | R2 | Dispersion | Deviation | Outliers | Standardized Residuals |
| --- | --- | --- | --- | --- | --- | --- |
| (Null model) Arrivals ~ 1 | 9366.8 | 0 | NS | NS | NS | Good |
| Arrivals ~ MoonPhase | 9338.3 | 0.13 | NS | NS | NS | Good |
| Arrivals ~ MoonPhase + TimeOfDay | 9133.7 | 0.57 | P=0.00 | NS | P=0.003 | Major quantile deviations detected |
| Arrivals ~ MoonPhase + TimeOfDay + MoonPhase*TimeOfDay | 8905.1 | 0.71 | NS | P=0.046 | NS | Minor quantile deviations detected |
| Arrivals ~ MoonPhase + TimeOfDay + LunarCycle | 9132.8 | 0.57 | P=0.0 | NS | NS | Major quantile deviations detected |
| Arrivals ~ MoonPhase + TimeOfDay + LunarCycle + MoonPhase*TimeOfDay | 8906.1 | 0.71 | NS | NS | NS | Major quantile deviations detected |
| Arrivals ~ MoonPhase + TimeOfDay + LunarCycle + LunarCycle*TimeOfDay | 9083.0 | 0.59 | P=0.0 | NS | P=0.018 | Major quantile deviations detected |
| Arrivals ~ MoonPhase + TimeOfDay + LunarCycle + MoonPhase*TimeOfDay + LunarCycle*TimeOfDay | **8874.4** | **0.71** | **NS** | **NS** | **NS** | **Minor quantile deviations detected** |

**S1 Table C. Estimated marginal means for interaction between moon phase and diel time for model of effects of moon phase, lunar cycle, diel time, and interactions on total arrivals.**

| Moon Phase | Diel Time | Estimated Marginal Mean | Standard Error | Df | Lower Confidence Interval | Upper Confidence Interval |
| --- | --- | --- | --- | --- | --- | --- |
| First quarter | Day | 4.54 | 0.163 | 807 | 4.22 | 4.85 |
| Full moon | Day | 4.87 | 0.112 | 807 | 4.65 | 5.09 |
| Third quarter | Day | 4.84 | 0.166 | 807 | 4.51 | 5.16 |
| New moon | Day | 4.47 | 0.168 | 807 | 4.14 | 4.80 |
| Waning crescent | Day | 4.83 | 0.160 | 807 | 4.51 | 5.14 |
| Waning gibbous | Day | 4.65 | 0.167 | 807 | 4.33 | 4.98 |
| Waxing crescent | Day | 4.53 | 0.171 | 807 | 4.19 | 4.86 |
| Waxing gibbous | Day | 4.90 | 0.168 | 807 | 4.57 | 5.23 |
| First quarter | Night | 2.06 | 0.188 | 807 | 1.69 | 2.43 |
| Full moon | Night | 4.78 | 0.130 | 807 | 4.52 | 5.03 |
| Third quarter | Night | 4.02 | 0.190 | 807 | 3.65 | 4.39 |
| New moon | Night | 1.68 | 0.193 | 807 | 1.30 | 2.06 |
| Waning crescent | Night | 1.48 | 0.191 | 807 | 1.11 | 1.86 |
| Waning gibbous | Night | 5.05 | 0.183 | 807 | 4.69 | 5.41 |
| Waxing crescent | Night | 1.61 | 0.204 | 807 | 1.21 | 2.01 |
| Waxing gibbous | Night | 4.38 | 0.177 | 807 | 4.04 | 4.73 |
| First quarter | Twilight | 5.76 | 0.329 | 807 | 5.12 | 6.41 |
| Full moon | Twilight | 5.80 | 0.265 | 807 | 5.28 | 6.33 |
| Third quarter | Twilight | 5.35 | 0.317 | 807 | 4.72 | 5.96 |
| New moon | Twilight | 5.70 | 0.315 | 807 | 5.08 | 6.32 |
| Waning crescent | Twilight | 5.89 | 0.345 | 807 | 5.21 | 6.57 |
| Waning gibbous | Twilight | 6.61 | 0.388 | 807 | 5.85 | 7.37 |
| Waxing crescent | Twilight | 5.58 | 0.366 | 807 | 4.86 | 6.30 |
| Waxing gibbous | Twilight | 5.59 | 0.345 | 807 | 5.25 | 6.60 |

**S1 Table D. Model selection for the effects of season and temperature on arrivals.** Final model selected is highlighted in bold.

| Model | AIC | R2 | Dispersion | Deviation | Outliers | Standardized Residuals |
| --- | --- | --- | --- | --- | --- | --- |
| (Null model) Arrivals ~ 1 | 13764 | 0 | NS | NS | NS | Good |
| (Null model with random intercept) Arrivals ~ 1 + (1\|ColonyID) | 13737 | 0.262 | NS | P=0 | NS | Good |
| Arrivals ~ Season + (1\|ColonyID) | 13734 | 0.169 | NS | P=0.01 | NS | Good |
| Arrivals ~ Season + MinTemp + (1\|ColonyID) | 13731 | 0.187 | NS | P=0.003 | NS | Minor Quantile deviations detected |
| Arrivals ~ Season + MinTemp + TimeOfDay + (1\|ColonyID) | 13590 | 0.396 | NS | P=0 | P=0.001 | Major Quantile deviations detected |
| Arrivals ~ Season + MinTemp + TimeOfDay + Season*TimeOfDay + (1\|ColonyID) | **13480** | **0.558** | **NS** | **P=0** | **P=9e-5** | **Major Quantile deviations detected** |

**S1 Table E. Pairwise differences in activity between diel times across all seasons.** Significant differences between activity rates during different diel times are illustrated using letters, where diel times with the same letter were not found to be significantly different but diel times with different letters were found to be significantly different.

| Season | Diel Time | Estimated Marginal Mean | Standard Error | Df | Lower Confidence Interval | Upper Confidence Interval | Group |
| --- | --- | --- | --- | --- | --- | --- | --- |
| Autumn | Day | 4.86 | 0.73 | 1237 | 3.42 | 6.29 | ABCDEFGHI |
| Autumn | Night | 2.98 | 0.54 | 1237 | 1.92 | 4.03 | ABCD |
| Autumn | Twilight | 4.35 | 0.57 | 1237 | 3.24 | 5.46 | EFGHI |
| Winter | Day | 4.08 | 0.63 | 1237 | 2.85 | 5.31 | BF |
| Winter | Night | 4.58 | 0.63 | 1237 | 3.35 | 5.81 | CG |
| Winter | Twilight | 5.40 | 0.64 | 1237 | 4.14 | 6.66 | DHI |
| Summer | Day | 4.87 | 0.62 | 1237 | 3.64 | 6.09 | CDGH |
| Summer | Night | 3.30 | 0.63 | 1237 | 2.07 | 4.53 | AE |
| Summer | Twilight | 5.85 | 0.64 | 1237 | 4.59 | 7.10 | I |
