# Supplementary material for: A hard day’s night: Patterns in the diurnal and nocturnal foraging behavior of Apis dorsata across lunar cycles and seasons
Source: PLoS One. 2021 Oct 22;16(10):e0258604. doi: 10.1371/journal.pone.0258604 (PMC8535376; doi:10.1371/journal.pone.0258604)
Supplement: S2 File — This file contains the results of a supplemental analysis comparing activity during the dawn twilight period to activity during the dusk twilight period across seasons. (A) The ANOVA output for the statistical analysis and (B) a summary figure of the results are included. (DOCX) [file pone.0258604.s002.docx]

**S2 Table A. ANOVA results for the analysis comparing dusk versus dawn activity across the study period.** There was no significant difference between activity at dawn and dusk, but a significant interaction was seen between the amount of activity at dawn or dusk and the season.

| Model Factor | F | DF | P-value |
| --- | --- | --- | --- |
| DawnOrDusk | 2.18 | 1 | 0.14 |
| Season | 5.89 | 2 | 0.053 |
| DawnOrDusk*Season | 30.59 | 2 | 2.28 e -7 |


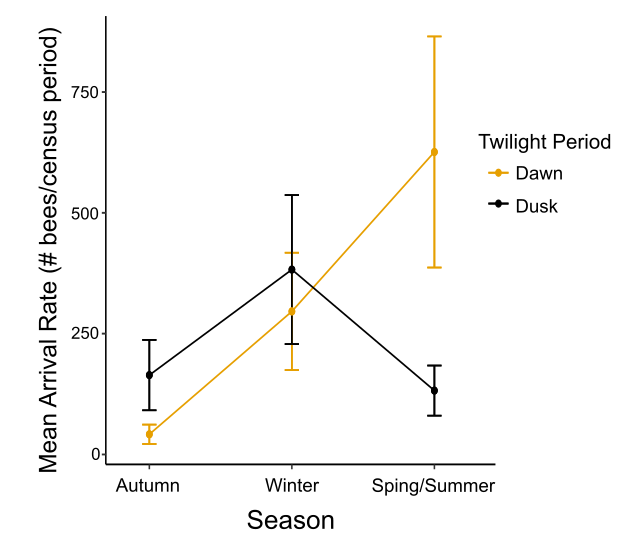


**S2 Fig B. Mean arrival rates at dawn and dusk.** Estimated marginal mean arrival rates during dawn versus during dusk across seasons are shown. Total activity did not significantly differ between dawn and dusk (P = 0.14), but the proportion of activity that occurred during dawn versus during dusk did significantly differ depending on the season (P = 2.28 e -7). In particular, arrival rates were higher during dawn than during dusk in spring/summer (P < 0.0001), but dawn and dusk arrival rates were the same in autumn (P = 0.066) and in winter (P = 0.96). ANOVA results can be found in S2 Table A.
