# Supplementary material for: A hard day’s night: Patterns in the diurnal and nocturnal foraging behavior of Apis dorsata across lunar cycles and seasons
Source: PLoS One. 2021 Oct 22;16(10):e0258604. doi: 10.1371/journal.pone.0258604 (PMC8535376; doi:10.1371/journal.pone.0258604)

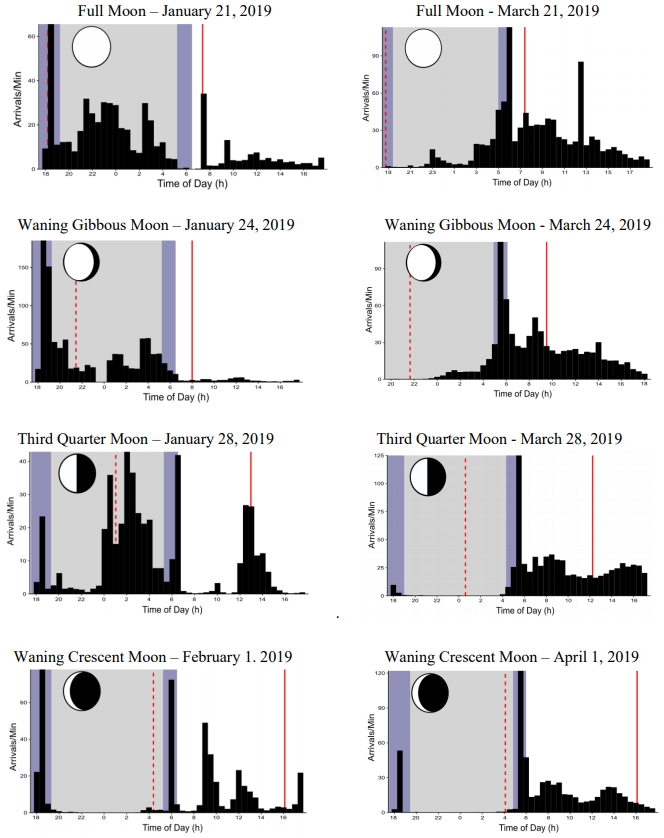


**S1 Fig. Daily foraging activity patterns of *A. dorsata*.** Daily foraging activity on each day recorded during the January to February (left) and March to April (right) lunar cycles are shown. Purple shaded regions indicate astronomical twilight, gray shaded regions indicate night, and white indicates daytime. The red dotted line indicated moonrise, while the solid red line indicates moonset. Activity tended to peak during the twilight periods and was only seen at night during the full moons and the weeks before and after them.

**S1 Fig. (cont’d)**


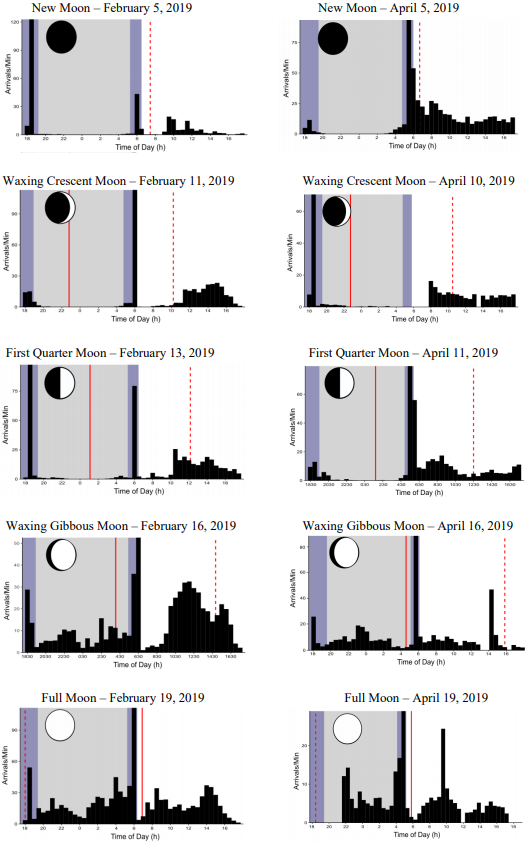

Supplement: S1 Fig — Daily foraging activity on each day recorded during the January to February (left) and March to April (right) lunar cycles are shown. Purple shaded regions indicate astronomical twilight, gray shaded regions indicate night, and white indicates daytime. The red dotted line indicated moonrise, while the solid red line indicates moonset. Activity tended to peak during the twilight periods and was only seen at night during the full moons and the weeks before and after them. (DOCX) [file pone.0258604.s003.docx]
